# Magnet-Assisted Anastomosis in Gastrointestinal Surgery: Current Evidence, Technical Considerations, and Barriers to Clinical Adoption

**DOI:** 10.7759/cureus.105175

**Published:** 2026-03-13

**Authors:** John Salib, Mark Salib, Aryan Kahlon, Shanu Sivakumar, Timothy J Stear

**Affiliations:** 1 School of Medicine, St. George's University, St. George's, GRD; 2 Department of General Surgery, Community First Medical Center, Chicago, USA; 3 Department of General Surgery, Resurrection Medical Center, Chicago, USA

**Keywords:** device-based anastomosis, endoscopic anastomosis, gastrointestinal anastomosis, gastrointestinal reconstruction, hybrid endoscopic-surgical technique, magnet-assisted anastomosis, magnetic anastomosis, magnetic compression anastomosis, minimally invasive surgery, sutureless anastomosis

## Abstract

Gastrointestinal (GI) anastomosis remains a cornerstone of abdominal surgery; however, anastomotic failure continues to represent a significant source of postoperative morbidity, mortality, and healthcare burden despite advances in suturing, stapling, and minimally invasive techniques. Magnet-assisted anastomosis (MAA) has recently re-emerged as an investigational, sutureless technique that utilizes sustained magnetic compression to induce localized ischemia, tissue necrosis, and subsequent biologic fusion of adjacent luminal surfaces without leaving permanent foreign material. Although early reports have demonstrated technical feasibility across select gastrointestinal, bariatric, and pediatric applications, the clinical evidence supporting MAA remains limited, heterogeneous, and largely confined to experimental studies and small clinical series. This qualitative narrative review critically synthesizes the available preclinical and early clinical literature to examine the mechanistic basis of MAA, current technical approaches, early feasibility outcomes, and the practical limitations that currently constrain broader clinical adoption. Particular attention is given to perioperative considerations unique to delayed anastomotic formation, including nutritional management, timing of luminal patency, device-related complications, and postoperative monitoring strategies. While preliminary studies report high technical success rates and successful formation of patent anastomoses in carefully selected patients, the existing literature remains constrained by small sample sizes, heterogeneous study designs, and limited long-term follow-up. Key questions regarding long-term durability, stricture formation, comparative leak risk, and standardized patient selection remain incompletely defined. By synthesizing the fragmented body of evidence on MAA, this review aims to clarify the current state of the field, identify critical knowledge gaps, and outline the research priorities needed to determine whether MAA can play a meaningful role in future gastrointestinal surgical practice.

## Introduction and background

Gastrointestinal (GI) anastomosis represents a fundamental component of abdominal surgery, enabling restoration of alimentary continuity following resection, diversion, or reconstructive procedures. Despite substantial technical refinement over the past century, including the transition from traditional hand-sewn techniques to modern stapling devices and minimally invasive approaches, anastomotic failure remains among the most consequential complications in GI surgery [1,2]. Reported leak rates vary considerably according to anatomic location and patient-specific risk factors, ranging from approximately 3% to over 15%, and are associated with significant morbidity, mortality, prolonged hospitalization, and long-term functional impairment [2]. Consequently, the optimization of anastomotic construction remains a central focus of surgical innovation.

Contemporary anastomotic techniques rely predominantly on sutures or mechanical stapling devices to achieve immediate tissue approximation and luminal continuity [3,4]. These approaches are well established, technically reproducible, and supported by extensive clinical experience. Nevertheless, they rely on precise tissue handling, adequate perfusion, and appropriate tension distribution to facilitate reliable healing while simultaneously introducing foreign material into the operative field [3,4]. Over the past several decades, numerous alternative concepts, including compression rings, biofragmentable anastomotic devices, and various mechanical coupling systems, have been investigated in an effort to simplify anastomotic construction or mitigate complications. However, the majority of these technologies have failed to achieve widespread clinical adoption, often due to technical limitations, inconsistent outcomes, or the absence of demonstrable advantages over established techniques [5].

Magnetic compression technology represents one such alternative approach that has periodically re-emerged in the surgical literature. Magnet-assisted anastomosis (MAA), also referred to as magnetic compression anastomosis (MCA), utilizes paired magnets positioned on opposing luminal surfaces to generate sustained compressive forces across intervening tissue layers [6-9]. This compression induces localized ischemia and controlled tissue necrosis, followed by progressive fusion of adjacent bowel walls and subsequent formation of a patent anastomotic channel [5-8]. Once tissue remodeling is complete, the magnets traverse the GI tract via natural excretion, thereby leaving no permanent implant within the operative site [6,8]. In contrast to conventional sutured or stapled anastomoses, which establish immediate luminal continuity, magnetic compression relies upon delayed biologic remodeling to achieve anastomotic maturation [7,9].

Early investigation of MAA primarily occurred in experimental models and pediatric surgical settings, particularly in the management of esophageal atresia, intestinal atresia, and biliary strictures [10,11]. More recently, advances in device engineering, endoscopic delivery platforms, and imaging guidance have renewed interest in selective adult applications, including bariatric and hepatobiliary procedures. Despite these developments, the clinical evidence supporting MAA remains limited in scope and is largely derived from small case series and early feasibility studies conducted in carefully selected patient populations [12-14].

As a result, several critical aspects of this technology remain incompletely defined. Available studies provide only limited insight into long-term durability, comparative leak risk, or the incidence of late complications such as stricture formation. Furthermore, the delayed nature of anastomotic maturation inherent to magnetic compression introduces distinct perioperative considerations, including nutritional management, timing of luminal patency, and postoperative monitoring strategies [7,9]. These practical challenges, together with the modest size of the current evidence base, may partially explain why MAA has not yet achieved routine clinical implementation within GI surgery.

Given the fragmented and heterogeneous nature of the existing literature, a critical synthesis of available data may help clarify both the potential applications and the current limitations of this technology. Accordingly, this qualitative narrative review examines the mechanistic principles underlying MAA, summarizes available experimental and early clinical evidence, and evaluates the practical barriers that have constrained broader clinical adoption. Particular emphasis is placed on the clinical contexts in which MAA has been investigated, including pediatric, biliary, and bariatric applications, to provide a balanced assessment of its current status and identify key priorities for future research.

History and background 

The conceptual basis of compression-mediated gastrointestinal anastomosis predates modern magnetic systems by more than a century. In 1892, John Benjamin Murphy introduced the Murphy Button, a nickel-plated mechanical coupler designed to achieve sutureless anastomosis through circumferential compression of opposing bowel walls [15-17]. Early surgical reports described reduced operative time and greater technical simplicity compared with hand-sewn techniques, and the device was initially adopted with considerable enthusiasm during a period marked by optimism regarding mechanical standardization in surgery [16,17]. However, clinical experience soon revealed important limitations. Reports of luminal obstruction, device retention, migration, and inconsistent anastomotic healing emerged as larger series accumulated, reflecting the inability of early mechanical systems to regulate compressive forces across variable tissue conditions in a consistent and predictable manner [16,17]. As surgical technique evolved and the emphasis on reproducibility and controlled operative outcomes increased, compression-based devices gradually fell out of favor, first replaced by refined suturing methods and later by mechanical stapling technologies [5,15-18].

Despite the decline of early compression devices, the biological principle underlying compression-mediated tissue fusion, ischemia-induced necrosis followed by controlled remodeling of adjacent luminal surfaces, remained conceptually sound [5,9]. Modern MAA represents a technological reinterpretation of this mechanism. Experimental studies have demonstrated that sustained compressive forces applied between adjacent hollow viscera can induce localized ischemia, tissue necrosis, and subsequent fusion of opposing luminal surfaces, thereby establishing the biological basis for MAA [19,20]. Unlike earlier rigid mechanical couplers, magnetic systems permit continuous and evenly distributed compressive forces without the need for a fixed mechanical locking mechanism [9,19].

Clinical interest in magnetic compression techniques initially emerged in pediatric surgery, particularly in the management of esophageal atresia, biliary strictures, and intestinal atresia, where magnets were used to approximate discontinuous segments without formal open reconstruction [10,11]. These early experiences demonstrated technical feasibility in anatomically challenging settings and stimulated further investigation into potential GI applications.

Advances in magnet technology, including the development of rare-earth neodymium magnets and improved biocompatible coatings, enabled more predictable compressive forces within compact device profiles suitable for minimally invasive and endoluminal delivery [7,9]. These technological developments coincided with the broader adoption of laparoscopic and endoscopic techniques, facilitating exploration of magnetic compression strategies in adult GI surgery [7,8].

In adult practice, early investigations have focused primarily on hepatobiliary and upper GI reconstruction, including gastrojejunostomy and choledochoenterostomy, with subsequent exploration in metabolic and bariatric procedures [6,13-14]. The introduction of purpose-built commercial systems such as Magnamosis and the MagDI™ system has further enabled structured feasibility studies, with early reports demonstrating high technical success rates and successful anastomotic formation in carefully selected patients [12-14]. Nevertheless, the available clinical evidence remains limited to small and heterogeneous cohorts, and robust comparative outcome data evaluating long-term durability, complication profiles, and performance relative to established anastomotic techniques remain lacking [2,7].

Viewed within this historical context, MAA can be understood not as an entirely novel concept, but rather as a contemporary evolution of compression-based anastomotic strategies first explored over a century ago. Advances in materials science, device engineering, and minimally invasive surgical platforms may address some of the limitations that historically constrained earlier compression devices. However, whether these technological developments will translate into meaningful clinical advantages over established sutured or stapled techniques remains uncertain and requires further systematic investigation.

## Review

Methods

**Figure 1:**
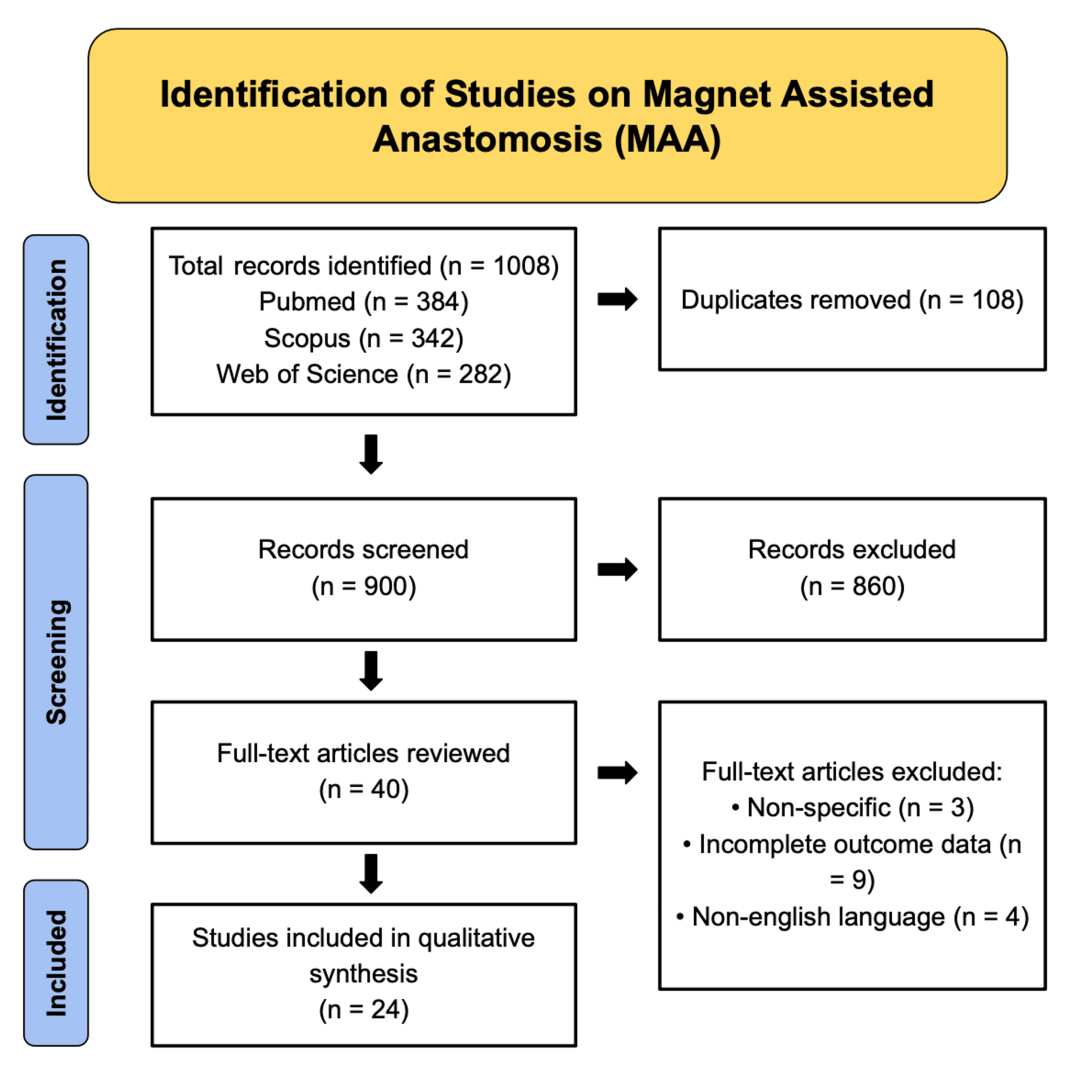
PRISMA flow diagram of study selection on magnet-assisted anastomosis Flowchart illustrating the systematic identification, screening, eligibility, and inclusion process for studies evaluating magnet-assisted anastomosis. A total of 1008 records were identified through database searching, including PubMed (n=384), Scopus (n=342), and Web of Science (n=282). After removal of 108 duplicates, 900 records were screened by title and abstract, of which 860 were excluded. Forty full-text articles were assessed for eligibility, and 16 were excluded due to non-specific relevance (n=3), incomplete outcome data (n=9), or articles that are non-English (n=4). A total of 24 studies were included in the final qualitative synthesis. PRISMA - Preferred Reporting Items for Systematic Reviews and Meta-Analyses

This study is a qualitative narrative review designed to synthesize the current evidence regarding the development, technical approaches, clinical applications, and safety profile of MAA within the GI tract. The review was conducted in accordance with methodological principles adapted from the Preferred Reporting Items for Systematic Reviews and Meta-Analyses (PRISMA) guidelines to enhance transparency and reproducibility (as depicted in Figure 1). A comprehensive literature search was performed from 2008 to 2026, capturing the evolution of MAA from early experimental models to contemporary clinical implementation. A total of 1008 records were identified through database searching, including PubMed (n=384), Scopus (n=342), and Web of Science (n=282). The search strategy incorporated both Medical Subject Headings (MeSH) and free-text keywords, including "magnet-assisted anastomosis", "magnetic compression anastomosis", "MCA", "magnetic rings", "magnetic coupling", "compression anastomosis", "sutureless anastomosis", "gastrointestinal magnetic anastomosis", "endoscopic magnetic anastomosis", "hybrid magnetic technique", and "minimally invasive anastomosis". Reference lists of relevant publications were manually screened to identify additional eligible studies.

Studies were eligible for inclusion if they evaluated magnet-assisted or magnetic compression anastomosis within the GI tract and reported clinical, procedural, or translational outcomes. Included study designs comprised randomized controlled trials, prospective and retrospective cohort studies, case-control studies, case series, technical reports, systematic reviews, meta-analyses, and narrative reviews. To ensure relevance, studies were required to report at least one of the following domains: procedural technique or deployment strategy (open, laparoscopic, endoscopic, or hybrid surgery), technical feasibility or success rates, anastomotic patency, leak rates, stricture formation, adverse events, reintervention rates, long-term durability, or device-specific considerations. Systematic reviews and meta-analyses were included when they provided pooled outcome data, comparative analyses, or broader evaluations of safety and efficacy. Narrative reviews were incorporated to contextualize device evolution, the mechanistic principles of compression-mediated healing, and emerging clinical applications. Preclinical animal studies were selectively included when they contributed foundational mechanistic insight, informed device engineering, or clarified biologic processes underlying compression-induced anastomotic formation.

Exclusion criteria were predefined to minimize heterogeneity unrelated to the scope of this review. Studies were excluded if they focused on non-GI magnetic applications, non-magnetic compression devices, purely surgical stapled or hand-sewn anastomoses without magnetic comparison or pharmacologic interventions unrelated to magnetic anastomosis. Conference abstracts without full-text publication, editorials without primary data or analytical synthesis, opinion pieces, letters to the editor lacking substantive outcome data, duplicate publications, and non-English articles were excluded. Studies with insufficient methodological detail or without reportable procedural or clinical outcomes were also excluded.

Five authors independently screened all retrieved titles and abstracts for relevance. Full-text articles meeting preliminary screening criteria were subsequently assessed for eligibility, and disagreements were resolved through consensus discussion among the investigative team. Data extraction included study design, sample size, anatomical target site, deployment technique, magnet configuration, compression characteristics, follow-up duration, technical success rates, patency outcomes, and complication profiles. For systematic reviews and meta-analyses, pooled estimates, heterogeneity measures, and comparative conclusions were recorded. The extracted data were compiled and are summarized in Table 1.

**Table 1:** Summary of key studies on MAA: study characteristics, indications, interventions, outcomes, and narrative insights This figure summarizes 24 studies evaluating MAA across experimental, clinical, and review-based designs. Preclinical and device-development studies demonstrate reliable anastomotic formation, adequate bursting strength, controlled compression, and favorable histologic healing. Clinical applications include biliary strictures, pediatric congenital conditions, and bariatric/metabolic procedures, with consistently high technical success, confirmed patency, and low complication rates. Comparative literature on conventional sutured and stapled techniques highlights ongoing risks of leaks and strictures, supporting the investigation of compression-based alternatives. Overall, current evidence suggests that MAA is a feasible, minimally invasive, sutureless technique with promising safety and efficacy across gastrointestinal indications [1-24]. MCA - magnetic compression anastomosis; MAA - magnet-assisted anastomosis; RYGB - Roux-en-Y gastric bypass

Year/ author	Study type	Population indication	Intervention	Comparator	Outcome measures	Results (baseline → follow-up)	Key findings
2023 Zhang et al. [1]	Preclinical animal experimental study	Beagle model undergoing total gastrectomy with digestive tract reconstruction	MCA for simultaneous esophagojejunostomy and jejunojejunostomy	Conventional sutured anastomosis	Anastomotic formation time, patency confirmation, bursting pressure, histologic healing (inflammation, fibrosis, mucosal regeneration), complications	Baseline: surgical reconstruction after gastrectomy → Follow-up: 4-8 weeks; successful anastomotic formation in all animals; bursting pressure comparable or higher than sutured controls; minimal inflammatory response and preserved mucosal continuity	Demonstrates the technical feasibility of MCA in complex digestive reconstruction with acceptable healing characteristics in preclinical models
2021 Zarnescu et al. [2]	Narrative review	Patients undergoing colorectal surgery	Conventional sutured or stapled anastomoses	None	Risk factors for anastomotic leakage	Identifies patient-related, technical, and disease-related risk factors contributing to leak rates	Provides context for ongoing efforts to improve anastomotic safety
2016 Jiang et al. [3]	Meta-analysis	Laparoscopic RYGB	Hand-sewn vs stapled gastrojejunal anastomosis	Hand-sewn vs mechanical	Leak rates, stricture rates, operative time	Mechanical techniques were associated with lower complication rates	Provides benchmark outcomes for conventional bariatric anastomosis
2014 Lee et al. [4]	Comparative clinical study	Laparoscopic RYGB patients	Multiple gastrojejunal anastomotic techniques	Technique comparison	Stricture rates, weight loss	Higher stricture rates were associated with certain techniques	Demonstrates the clinical consequences of anastomotic technique
2008 Kaidar-Person et al. [5]	Historical review	Evolution of compression anastomosis techniques	Mechanical compression devices	None	Historical outcomes and complications	Describes the development from the Murphy button to modern compression approaches	Establishes a conceptual foundation for compression-based anastomosis
2011 Jang et al. [6]	Clinical case series	Patients with refractory biliary anastomotic strictures following living donor liver transplantation	MCA recanalization using percutaneous or endoscopic magnet placement	Prior failed endoscopic therapy	Technical success, restoration of biliary drainage, complication rates	Baseline: obstructed biliary anastomoses → Follow-up weeks–months; recanalization successful in the majority of cases; improvement in cholestasis markers	Demonstrates the feasibility of MCA for complex biliary strictures after failure of conventional endoscopic management
2024 Zhang et al. [7]	Narrative review	GI strictures and obstruction treated endoscopically	Endoscopic magnetic compression techniques	None	Technical success rates, time to patency, complications	Literature review reports high success rates in selected biliary and esophageal indications; complication rates vary depending on anatomy	Highlights expanding endoscopic uses of MCA but emphasizes the heterogeneity of available data
2024 Ünal et al. [8]	Review	MCA procedures requiring interventional radiology support	Image-guided magnet delivery and positioning	None	Technical feasibility, procedural success	Multidisciplinary approaches enable magnet placement in anatomically challenging cases	Highlights the role of interventional radiology in facilitating MCA deployment
2022 Trujillo Loli et al. [9]	Narrative review	Digestive surgery requiring anastomosis	Magnetic compression techniques	None	Indications, technical principles, complications	Literature overview describing multiple GI applications	Summarizes principles and potential indications for magnetic compression
2023 Kotlovsky et al. [10]	Narrative review with case synthesis	Pediatric surgical indications, including esophageal atresia, Hirschsprung disease, ureteral and urethral strictures (reported cohort ~86 children)	Magnetic compression techniques were used for recanalization or anastomosis	Historical surgical or endoscopic approaches	Technical success, complications, need for reintervention, long-term patency	Review of ~87 procedures; reported success rates ~80–90% depending on indication; failures related to magnet displacement, insufficient compression force, or tissue interposition	Suggests MCA may be effective in selected pediatric indications while highlighting limitations in device design and the need for further refinement
2022 Holler et al. [11]	Systematic review (PRISMA)	Pediatric long-gap esophageal atresia	Magnetic compression esophageal anastomosis	Historical techniques	Success rates, strictures, need for dilation	Stricture formation was common; newer approaches may reduce intervention burden	Demonstrates the technical challenges of esophageal MCA
2023 Gagner et al. [12]	First-in-human feasibility study	Adults with severe obesity ± type 2 diabetes undergoing metabolic diversion	Side-to-side magnetic compression duodeno-ileostomy using the magnet anastomosis system	None	Technical success, magnet deployment, time to anastomosis formation, adverse events, metabolic outcomes	Baseline: BMI ≥35 cohort → Follow-up months; successful magnet placement and coupling in all patients; patent anastomosis confirmed; early weight loss and metabolic improvements reported	Provides initial human feasibility data demonstrating that magnet-assisted duodeno-ileostomy can be successfully performed in a small bariatric cohort
2023 Gagner et al. [13]	Multicenter clinical study	Adults undergoing sleeve gastrectomy requiring diversion	Magnetic side-to-side duodeno-ileostomy	None	Technical success, weight loss outcomes, safety	Early follow-up shows successful magnet deployment across centers	Early multicenter experience suggests the feasibility of magnetic diversion
2026 Bhandari et al. [14]	Prospective multicenter clinical study	Patients undergoing RYGB	Immediately patent magnetic jejuno-jejunal anastomosis (IMPA-JJ)	None	Safety, patency, short-term complications	Immediate patency achieved with acceptable early safety outcomes	Demonstrates the feasibility of immediate-patency magnetic anastomosis
1892 Murphy [15]	Historical surgical report	Gastrointestinal anastomosis procedures	Murphy button compression device	Sutured anastomosis	Technical feasibility and postoperative recovery	Early reports describing the sutureless compression anastomosis technique	Foundational concept for compression-based GI anastomosis
1893 Cordier [16]	Historical case report	Acute intestinal obstruction	Anastomosis using the Murphy button	None	Clinical recovery and postoperative outcomes	Successful intestinal reconstruction reported	Early clinical application of compression anastomosis
1903 Dunn [17]	Clinical series	Intestinal resections	Murphy button vs sutured anastomosis	Sutured technique	Complications and postoperative outcomes	Mixed outcomes with some complications, including obstruction	Demonstrates early advantages and limitations of compression devices
2022 Do et al. [18]	Case report with procedural video	Post-cholecystectomy biliary stricture	Endoscopic MCA recanalization	None	Technical success, patency confirmation	Successful magnet coupling and recanalization were achieved endoscopically	Demonstrates technical feasibility in a single biliary case
2020 Chen et al. [19]	Device development and experimental validation	Preclinical intestinal anastomosis models	Fedora-type MCA device designed to optimize compression force distribution	Conventional ring-shaped MCA devices	Compression pressure uniformity, anastomotic diameter, tissue necrosis, stenosis risk	Baseline device deployment → Follow-up days–weeks; device generated a larger and more uniform anastomotic lumen with reduced uneven pressure	Device design modifications may improve compression control and anastomotic geometry
2024 Zhang et al. [20]	Experimental study	Digestive tract tissue models under varying tension	MCA under different tension conditions	None	Patency, necrosis, histologic healing	Increased tissue tension was associated with impaired healing and greater necrosis	Highlights the importance of mechanical conditions for successful MCA
2026 Yeung et al. [21]	Animal experimental study	Porcine model with metabolic diversion	Magnetic compression duodeno-ileal diversion	None	Metabolic parameters, anastomotic formation	Successful diversion with metabolic improvements in an animal model	Demonstrates potential metabolic applications in a preclinical setting
2026 Chiappetta et al. [22]	Multicenter clinical report	Patients with obesity undergoing magnetic duodeno-ileal diversion	Magnetic compression anastomosis system (MagDI™)	None	Technical success and early outcomes	Early experience confirms the feasibility of device deployment	Provides additional clinical experience with magnet-assisted diversion
2026 Desouky et al. [23]	Systematic review and meta-analysis	Benign and malignant biliary obstruction	MCA recanalization techniques	None	Technical success, recurrence, complications	Pooled success rates frequently >90% with low recurrence	Largest pooled analysis evaluating MCA for biliary obstruction
2026 Jang et al. [24]	Retrospective long-term study	Completely obstructed benign biliary strictures	MCA recanalization with stent support	None	Technical success, recurrence, long-term patency	Technical success >90%; recurrence manageable with non-surgical treatment	Demonstrates long-term durability in biliary applications

Given the substantial heterogeneity in study design, anatomical indications, device configurations, and reported outcome measures, quantitative meta-analysis was not performed. Instead, findings were synthesized using a structured qualitative narrative approach integrating primary clinical data with higher-level evidence from systematic analyses and narrative reviews. The literature was organized according to device evolution, deployment strategies, mechanisms of compression-mediated healing, clinical efficacy, safety outcomes, and emerging applications, thereby providing a comprehensive and clinically relevant overview of the current landscape of magnet-assisted GI anastomosis.

Principles of magnet-assisted anastomosis

Magnet-assisted anastomosis is based on the controlled application of sustained compressive forces across adjacent segments of gastrointestinal (GI) tissue to induce localized ischemia, tissue necrosis, and subsequent fusion of opposing luminal surfaces [9,19,20]. In contrast to conventional anastomotic techniques that rely on immediate mechanical approximation using sutures or stapling devices, MAA leverages biologically mediated tissue remodeling to ultimately establish luminal continuity without leaving permanent foreign material in situ [7,9].

The fundamental principle involves the placement of paired magnets on opposing sides of the intended tissue interface, typically within adjacent bowel segments or hollow viscera. Magnetic attraction generates a consistent circumferential compressive force that maintains precise tissue apposition [9,19]. This sustained compression leads to progressive ischemia of the intervening tissue, resulting in controlled necrosis and sloughing of the compressed tissue layer. Concurrently, surrounding viable tissue undergoes reparative remodeling, eventually forming a patent anastomotic channel between the two luminal surfaces [9,19,20].

A defining characteristic of MAA is the delayed formation of the anastomosis. Unlike sutured or stapled techniques, which create immediate structural continuity of the GI tract, magnetic compression anastomosis relies on a gradual biological process that typically unfolds over several days as necrotic tissue separates and mucosal continuity is re-established [7,9]. Once an adequate anastomotic tract has formed, the coupled magnets pass spontaneously through the GI tract, thereby eliminating the need for permanent implant retention and reducing long-term foreign-body exposure [7,8].

However, this delayed maturation of the anastomosis represents more than a technical distinction; it introduces a set of practical perioperative challenges that may partly explain the limited clinical adoption of this technique. Because luminal continuity is not established immediately, patients may experience a temporary period in which GI flow remains restricted, necessitating careful consideration of nutritional support, decompression strategies, and postoperative monitoring. Furthermore, the interval between device placement and complete anastomotic maturation introduces theoretical concerns regarding leakage, obstruction, or incomplete anastomotic formation during the early postoperative period [7,9].

The successful application of MAA therefore depends not only on device placement but also on several critical technical and biological parameters, including accurate magnet alignment, appropriate compressive force, adequate tissue perfusion, and the absence of significant inflammation or contamination at the intended anastomotic site [9,19,20]. Excessive compressive force may result in tissue injury beyond the intended anastomotic zone, whereas insufficient compression may fail to achieve complete tissue necrosis and subsequent luminal fusion [19,20]. Device design and procedural technique are therefore central determinants of both safety and efficacy [9,19].

From a mechanistic standpoint, MAA differs fundamentally from conventional anastomotic techniques not only in the method of tissue approximation but also in the temporal dynamics of anastomotic healing. Whereas sutured and stapled anastomoses provide immediate structural continuity, allowing early restoration of GI function, magnetic compression anastomosis requires a period of biologic remodeling before achieving functional patency [7,9]. This distinction has important implications for postoperative management, including decisions regarding enteral feeding, imaging surveillance, and patient selection. As such, the delayed nature of MAA remains one of the principal factors that must be addressed before broader clinical adoption can be realistically considered [7,9].

Indications and patient selection

Magnet-assisted anastomosis should presently be regarded as an investigational technique applicable primarily to carefully selected patients undergoing elective or semi-elective GI reconstruction under controlled operative conditions [12-14]. Available clinical experience remains limited, and most reported cases involve highly selected populations in which both the anatomic configuration and physiologic environment are favorable for controlled magnetic compression. As a result, current evidence does not support broad application across GI surgery but rather suggests that MAA may be most appropriately considered within a small number of narrowly defined clinical contexts.

Existing reports indicate that ideal candidates are those with well-perfused, non-inflamed bowel segments and anatomical configurations that permit precise magnet alignment and stable circumferential tissue apposition [9,19,20]. MAA achieves luminal continuity through delayed, biologically mediated tissue fusion rather than immediate mechanical approximation and is therefore most suitable for procedures in which delayed anastomotic maturation can be safely accommodated and perioperative nutritional strategies can be anticipated and managed in advance [7,9]. In the limited clinical literature available to date, applications have been concentrated primarily in upper gastrointestinal and hepatobiliary contexts, including gastrojejunostomy following distal gastrectomy, bariatric side-to-side duodeno-ileostomy configurations, and select biliary-enteric reconstructions, where controlled alignment of luminal structures can be achieved under elective operative conditions [6,12-14].

Successful implementation of MAA depends not only on patient selection but also on favorable physiologic and technical parameters. Clean or clean-contaminated operative fields are generally preferred, as significant inflammation or contamination may impair microvascular perfusion, increase tissue edema, and disrupt uniform circumferential compression, thereby compromising the controlled compression-necrosis-fusion sequence required for reliable anastomotic formation [9,19,20]. Adequate vascular perfusion is similarly essential, as the reparative remodeling process that ultimately establishes the anastomotic tract depends upon preserved microvascular integrity [9,20].

Conversely, several clinical scenarios represent clear contraindications. MAA is not appropriate in situations requiring immediate restoration of GI continuity, such as emergent operations or hemodynamically unstable patients, as the technique inherently relies on delayed anastomotic maturation [7,9]. Conditions associated with compromised tissue perfusion, including severe inflammation, ischemia, edema, or prior radiation injury, may disrupt the tightly regulated compression-necrosis-fusion sequence required for reliable healing and therefore represent unfavorable settings for magnetic compression techniques [9,19,20]. Likewise, gross intra-abdominal contamination, peritonitis, or uncontrolled infection introduces substantial biologic uncertainty during the interval preceding complete tissue integration and may increase the risk of septic complications [2,7].

Technical considerations also impose important constraints. Anatomical factors that preclude accurate magnet alignment, such as pronounced bowel angulation, luminal diameter mismatch, dense adhesions, or distorted postoperative anatomy, may generate asymmetric compressive forces and predispose to incomplete anastomotic formation or stricture development [19,20]. Physiologic limitations must also be considered, particularly in patients unable to tolerate delayed enteral intake due to severe malnutrition, lack of alternative nutritional access, or the clinical necessity for early feeding [7,9].

Taken together, these constraints underscore that the current clinical applicability of MAA remains relatively narrow. Until larger comparative studies clarify long-term outcomes, complication profiles, and standardized selection criteria, its use should likely remain limited to carefully selected elective cases in which both biologic and technical conditions are optimized. Within this restricted framework, MAA may represent a potential adjunct for specific reconstructive scenarios rather than a broadly applicable alternative to established anastomotic techniques.

Surgical techniques and approaches

The technical efficacy and clinical success of MAA are predicated on a mechanobiological process that fundamentally distinguishes it from the immediate mechanical fixation of suture-based or stapled closures [7,12]. Rather than relying on traumatic tissue penetration, MAA utilizes a process of compression-mediated ischemic remodeling to establish luminal continuity. Once the paired magnetic elements are coupled, they exert a constant circumferential compressive force across the intervening tissue interface [19,20]. This sustained pressure induces localized ischemia and controlled necrosis within the compressed tissue layer. Simultaneously, at the peripheral margins where compressive forces dissipate, an inflammatory and reparative response facilitates fibroblast proliferation and serosal-to-serosal adhesion [9,19,20]. Through this coordinated process of tissue necrosis and peripheral healing, the surrounding viable tissue margins unite through biologic remodeling, ultimately forming an epithelialized conduit as the necrotic tissue bridge separates and the magnetic device is liberated into the newly formed lumen [7,9,20].

Achieving this biologic fusion requires careful preoperative evaluation and precise anatomical planning to ensure the integrity of the intended anastomotic site. Assessment focuses on identifying well-perfused, non-inflamed bowel segments, as the success of the remodeling process is highly dependent on baseline tissue perfusion and structural integrity [19,20]. Clinicians frequently utilize cross-sectional imaging and endoscopic evaluation to define the spatial orientation of the target luminal structures and confirm the absence of transmural inflammation or malignancy that could impair stable compression-mediated fusion [7,19]. Intraoperative adjuncts such as indocyanine green (ICG) fluorescence angiography may also be employed to evaluate tissue perfusion at the intended interface and confirm the physiologic suitability of the anastomotic site [7]. These preparatory steps are important to minimize the risk of complications such as delayed stricture formation or incomplete anastomotic maturation.

**Figure 2:**
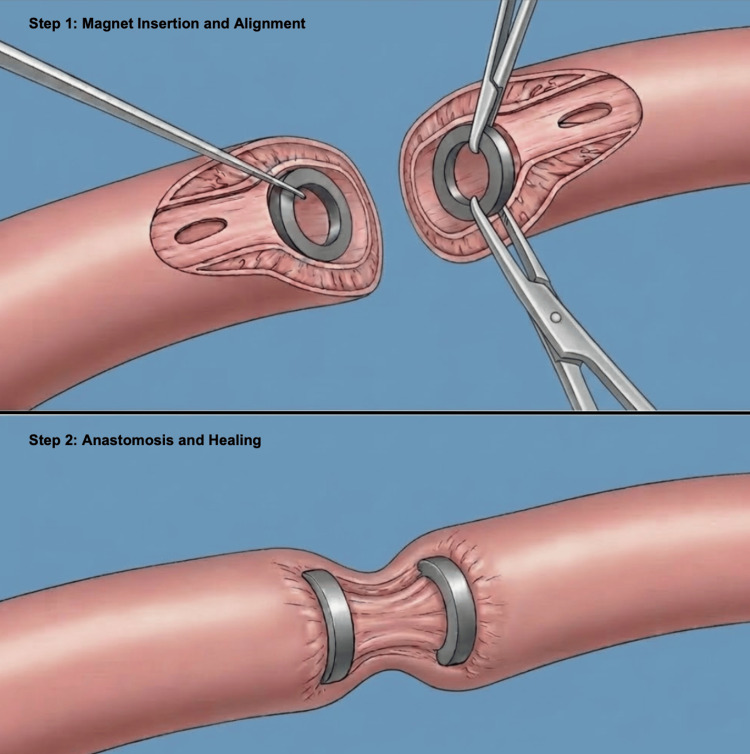
Laparoscopic/open surgical magnet-assisted anastomosis Schematic illustration of magnet-assisted anastomosis performed via laparoscopic or open surgical access. Step 1: Controlled enterotomies are created in the respective bowel segments to permit intraluminal insertion of paired magnetic rings. Correct polarity is confirmed, and the magnets are positioned flush against opposing mucosal surfaces to achieve circumferential tissue apposition. Direct visual and tactile feedback confirms accurate serosal alignment. Step 2: Magnetic attraction compresses the intervening tissue, establishing a stable approximation and initiating biologic anastomotic formation. The access enterotomies are subsequently either closed or incorporated into the final anastomotic configuration, depending on operative design [7,18]. Figure designed and created by the authors using BioRender.

In laparoscopic or open surgical approaches (as depicted in Figure 2), the procedure begins with the creation of controlled enterotomies in the respective bowel segments to facilitate intraluminal insertion of the magnetic elements. Surgeons must ensure correct magnetic polarity and precise positioning of the devices against the opposing mucosal surfaces in order to establish uniform circumferential compression across the target interface [7,18]. This approach allows direct visual confirmation of serosal alignment and tension-free positioning, both of which are important for stable anastomotic formation. Once magnetic attraction approximates the bowel segments, the access enterotomies are either closed primarily or incorporated into the final anastomotic configuration, depending on the procedural design [7,18].

**Figure 3:**
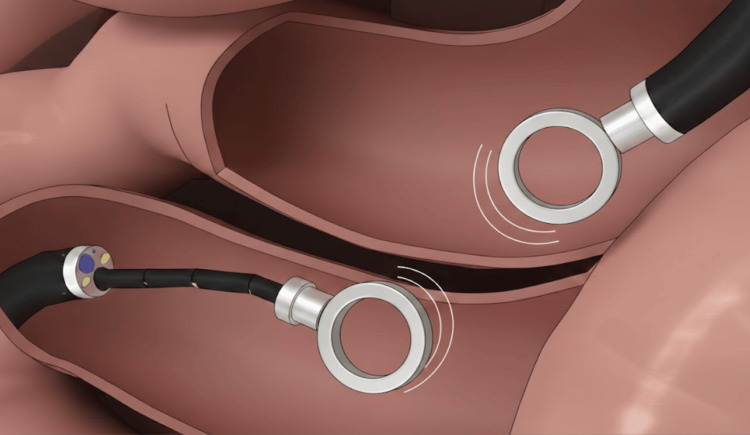
Fully endoscopic magnet-assisted anastomosis deployment Schematic depiction of a purely endoscopic approach to magnet-assisted anastomosis. Paired magnetic rings are delivered through large-caliber endoscopic working channels or dedicated catheter systems and advanced to the target site under direct visualization. Fluoroscopic guidance confirms accurate transmural alignment prior to magnetic coupling. This minimally invasive approach eliminates the need for surgical enterotomies but requires advanced endoscopic expertise to ensure precise axial alignment and avoid compression of adjacent structures [13,18]. Figure designed and created by authors using BioRender.

Endoscopic deployment (as depicted in Figure 3) represents a less invasive alternative involving the delivery of magnets through large-caliber working channels or specialized catheter systems. This technique relies on the combined use of direct endoscopic visualization and fluoroscopic imaging to guide device positioning and confirm accurate transmural alignment prior to magnetic coupling. Although this approach avoids surgical enterotomies and may reduce procedural invasiveness, it requires advanced endoscopic expertise and careful imaging confirmation to prevent asymmetric compression or inadvertent entrapment of adjacent structures [13,18]. Achieving reliable axial alignment across opposing bowel walls remains technically challenging when tactile feedback is limited.

Hybrid techniques integrate the strengths of both approaches by combining laparoscopic visualization with endoscopic delivery to improve spatial orientation in anatomically complex regions [9]. This strategy can be particularly useful in locations such as the gastroesophageal junction or deep pelvic cavity, where direct surgical exposure is limited and precise intraluminal positioning is technically demanding. Through coordinated collaboration between surgeon and endoscopist, laparoscopic visualization provides external anatomical orientation while endoscopic guidance facilitates accurate intraluminal magnet deployment [9].

Across all deployment strategies, the magnitude and distribution of compressive force generated by the magnetic elements remain key determinants of successful anastomotic formation [19,20]. Uniform circumferential compression is necessary to promote controlled tissue necrosis and consistent remodeling, whereas excessive or asymmetric compression may compromise anastomotic integrity or lead to eccentric healing and luminal narrowing [9,19,20]. For this reason, confirmation of magnet position through intraoperative imaging or endoscopic visualization is considered an essential technical step.

Postoperative management must account for the delayed formation of luminal continuity inherent to magnetic compression techniques. During this interval, patients may require nutritional support and radiographic or endoscopic confirmation of anastomotic patency before resuming oral intake [7,8]. The process is completed when the magnets detach from the anastomotic site and are passed spontaneously through the GI tract, indicating successful establishment of functional luminal continuity [9,20].

Clinical outcomes

Contemporary clinical experience with MAA in bariatric surgery remains limited and is derived primarily from small, early-phase cohorts designed to evaluate procedural feasibility rather than clinical effectiveness [12,13,20]. The first-in-human implementation of side-to-side magnetic compression anastomosis reported a 100% technical success rate (n=5) for magnet deployment, device expulsion, and formation of a patent anastomosis [12,23]. Although these findings demonstrate procedural feasibility, the extremely small cohort size substantially limits external validity and precludes meaningful assessment of reproducibility, complication rates, or procedural reliability across broader surgical populations.

In this initial experience, magnet expulsion occurred between 22 and 92 days following deployment, reflecting variability in compression-mediated tissue remodeling and anastomotic maturation [12,24]. No device-related adverse events were reported within the first 30 days. However, procedure-related complications, including mucosal and serosal injury, wound pain, and intra-abdominal hematoma, were observed, with additional delayed adverse events managed non-operatively during extended follow-up through 12 months [12]. Given the delayed nature of compression-mediated anastomosis formation, these findings highlight the importance of structured postoperative surveillance during the prolonged remodeling period characteristic of magnetic compression techniques.

Within bariatric applications, early metabolic outcomes following magnetic compression duodeno-ileostomy have been reported in small exploratory cohorts. Twelve-month outcomes from the initial feasibility study demonstrated 34±1.4% total weight loss, accompanied by reductions in HbA1c and fasting glucose levels [12]. Subsequent multinational experience involving 24 patients reported similar improvements in weight and glycemic parameters at six months, with persistence of these changes in a subset of patients at 12 months [13]. In this cohort, 85% of patients with type 2 diabetes receiving pharmacologic therapy at baseline discontinued antidiabetic medications by six months [13,14]. However, these findings must be interpreted cautiously, given the small sample sizes, potential selection bias, cohort attrition, and absence of comparator groups. At present, these observations should therefore be regarded as hypothesis-generating rather than evidence of established metabolic efficacy.

Experience from other clinical contexts provides additional insight into the technical behavior of magnetic compression anastomoses. In pediatric esophageal atresia repair, earlier magnetic compression approaches were associated with high stricture rates and frequent post-procedural dilation requirements. More recent refinements incorporating improved tissue alignment and controlled compression have demonstrated reductions in the need for subsequent interventions, although stricture incidence remains relatively high [11]. Although these studies arise from different anatomical and clinical contexts, they illustrate a critical technical principle relevant to MAA: successful magnetic anastomosis depends not only on compressive force but also on tissue alignment, luminal geometry, and tension distribution. These factors likely play an important role in determining long-term patency and anastomotic durability.

More recently, preliminary multicenter experience using the MagDI™ magnetic compression system has expanded clinical evaluation of magnet-mediated duodeno-ileal diversion beyond the initial first-in-human feasibility cohorts [13,21,22]. Early reports continue to demonstrate high technical success rates for magnet deployment, anastomosis formation, and device expulsion, supporting the procedural reproducibility of magnetic compression under controlled conditions. However, these data remain derived from small, non-comparative cohorts with limited follow-up and therefore cannot establish clinical effectiveness or define the role of magnetic compression techniques relative to established bariatric procedures [12,13,20].

Parallel preclinical investigations have explored the metabolic consequences of magnet-created intestinal diversion. For example, experimental duodeno-ileal diversion using magnetic compression in a porcine diabetes model demonstrated improvements in glycemic parameters following successful anastomosis formation [9,21,22]. While these findings provide mechanistic support for the metabolic rationale underlying magnetic bypass physiology, they remain preclinical and further underscore the incompletely characterized therapeutic implications of magnet-mediated diversion in human populations [21].

Taken together (as depicted in Table 2), the currently available clinical evidence suggests that MAA can achieve technical feasibility and successful anastomosis formation in carefully selected patients [12,13,20]. However, the existing evidence base remains preliminary and consists largely of small, uncontrolled cohorts with limited longitudinal follow-up. Consequently, the available literature is insufficient to establish clinical effectiveness or support routine clinical application in bariatric surgery. At present, MAA should therefore be regarded as an investigational reconstructive strategy. Clarifying its potential clinical role will require standardized procedural protocols, adequately powered comparative trials, and long-term evaluation of both anastomotic durability and metabolic outcomes.

**Table 2:** Early clinical and experimental evidence evaluating magnet-assisted anastomosis Summary of experimental and early clinical studies investigating magnet-assisted anastomosis. Reported variables include study design, indication, sample size, technical feasibility, clinical or metabolic outcomes, safety observations, and key methodological limitations. The current evidence base remains preliminary and consists primarily of small exploratory cohorts, early feasibility studies, and limited preclinical investigations with short- to mid-term follow-up [9,11-13,20-22].

Study/ reference	Study design/ setting	Clinical indication	Sample size	Technical outcomes	Clinical/ metabolic outcomes	Safety findings	Follow-up	Key limitations
First-in-human pilot study [12]	Prospective single-center feasibility study	Magnetic side-to-side duodeno-ileostomy for metabolic diversion	5	100% successful magnet deployment, coupling, and anastomosis formation; device expulsion 22–92 days	34±1.4% total weight loss at 12 months; HbA1c reduced from 6.8% to 4.8%; fasting glucose decreased from 134 to 87 mg/dL	No device-related adverse events within 30 days; procedure-related complications included mucosal/ serosal injury, wound pain, and intra-abdominal hematoma	Up to 12 months	Extremely small cohort; absence of comparator group; limited generalizability
Multinational observational cohort [13]	Multicenter observational study	Magnetic duodeno-ileostomy for obesity and metabolic disease	24	Technical feasibility was reproduced across multiple centers with successful magnet deployment and anastomosis formation	Mean weight reduction -34.2 kg at six months; sustained metabolic improvements at 12 months in subset; HbA1c reduction -1.1% at six months and -2.0% at 12 months; 85% discontinued diabetes medications by six months	No major early device-related adverse events reported	Up to 12 months (subset follow-up)	Non-comparative design; cohort attrition during follow-up; long-term durability uncertain
Pediatric magnetic compression refinement [11]	Comparative historical clinical analysis	Esophageal atresia repair using magnetic compression anastomosis	4	Improved pre-anastomotic pouch approximation (100% vs 21%) compared with earlier techniques	Not applicable	Stricture formation remains high (95-100%); dilation burden reduced (4.25 vs 9.5 dilations); no postoperative stent placement or surgical revision required	Short- to mid-term follow-up	Different anatomical and clinical populations; limited applicability to adult gastrointestinal reconstruction
Early MagDI™ system clinical experience [13,20–22]	Early multicenter feasibility reports	Magnetic duodeno-ileal diversion using MagDI™ compression system	Not reported	High procedural success for magnet deployment, coupling, and anastomosis formation was reported across early clinical series	Early weight loss and glycemic improvement trends were consistent with metabolic diversion physiology	Acceptable early safety profile; limited device-related complications reported	Early follow-up periods	Small uncontrolled cohorts; limited longitudinal data; heterogeneous reporting
Preclinical metabolic diversion model [9,21,22]	Experimental porcine model	Magnetic compression duodeno-ileal diversion	Not reported	Successful magnet coupling and anastomosis formation in an experimental model	Improved glycemic parameters following intestinal diversion	No major procedural complications were reported in the experimental setting	Experimental follow-up	Preclinical model; findings require validation in human clinical populations

Comparison with conventional anastomotic techniques

Magnet-assisted anastomosis represents a fundamentally different approach to GI reconstruction compared with conventional hand-sewn or stapled techniques. Traditional anastomoses establish immediate luminal continuity through direct tissue approximation secured with sutures or mechanical staples, allowing early restoration of GI function and enteral nutrition. In contrast, MAA relies on paired magnets that generate controlled circumferential compression, producing localized ischemia and tissue necrosis followed by gradual biologic fusion over several days to weeks. This delayed process introduces distinct perioperative considerations, including temporary restriction of enteral intake, surveillance for magnet position and eventual device expulsion, and dependence on adequate tissue alignment, perfusion, and mechanical tension to achieve a durable anastomosis [7,13,20].

From a technical perspective, MAA eliminates the need for suturing or stapling at the anastomotic interface and does not leave permanent intraluminal foreign material following device expulsion. These characteristics have prompted interest in its potential application within minimally invasive or anatomically constrained operative fields [7,18]. However, these theoretical advantages must be balanced against procedural sensitivities unique to compression-mediated anastomosis formation. Successful outcomes depend heavily on accurate magnet positioning and consistent tissue apposition; malalignment, distorted anatomy, or excessive tension may lead to asymmetric compression, stricture formation, or incomplete anastomotic fusion [7,13,20]. Both experimental and early clinical observations suggest that magnet geometry, compressive force, and tissue configuration influence anastomotic maturation, highlighting technical dependencies that differ from those associated with conventional stapled or hand-sewn reconstruction [19,20,22].

Comparative clinical evaluation between MAA and established anastomotic techniques remains limited. Early feasibility studies demonstrate reliable magnet deployment and successful anastomosis formation in small, carefully selected cohorts, but these investigations were not designed to evaluate comparative effectiveness [12,13]. Operative durations reported for magnetic duodeno-ileal diversion fall within ranges described for established bariatric procedures such as single-anastomosis duodeno-ileal bypass with sleeve gastrectomy (SADI-S) and duodenal switch (DS). However, meaningful comparisons are difficult given differences in patient selection, operative technique, and study design [13]. Similarly, reported weight loss and metabolic improvements following magnet-mediated diversion appear broadly consistent with outcomes observed after conventional bariatric operations, although these observations derive from small, uncontrolled cohorts and should therefore be interpreted cautiously [12,13]. At present, available evidence is insufficient to determine whether MAA offers measurable clinical advantages in safety, durability, or metabolic effectiveness relative to established reconstructive methods.

Experience from other clinical contexts further illustrates both the potential applicability and limitations of magnetic compression techniques. In pediatric esophageal atresia repair, early magnetic anastomosis approaches were associated with relatively high rates of postoperative stricture requiring repeated dilations. Subsequent refinements emphasizing improved tissue approximation and alignment have reduced intervention burden, although stricture formation remains common [11]. These observations reinforce that compression-mediated anastomosis formation is highly sensitive to tissue alignment, luminal geometry, and mechanical tension, factors that are also likely to influence long-term outcomes in adult GI reconstruction [19,20].

Taken together, as summarized in Table 3, MAA represents a mechanistically distinct reconstructive strategy that differs fundamentally from conventional hand-sewn and stapled anastomotic techniques. While early feasibility studies demonstrate that magnet-mediated anastomosis formation is technically achievable, current clinical evidence remains preliminary and does not establish superiority or equivalence relative to established surgical approaches. As with many emerging surgical technologies, it remains uncertain whether MAA will ultimately achieve widespread clinical adoption or remain limited to specialized or investigational applications. Definitive evaluation will require well-designed comparative trials with standardized operative protocols and long-term follow-up to assess anastomotic durability, metabolic outcomes, and perioperative morbidity. Until such data are available, MAA should be considered an investigational adjunct rather than a validated alternative to conventional GI anastomotic techniques [7,12,13,20].

**Table 3:** Comparison of magnet-assisted anastomosis and conventional gastrointestinal anastomotic techniques Comparison of mechanistic principles, technical characteristics, perioperative considerations, and current evidence supporting magnet-assisted anastomosis (MAA) relative to conventional hand-sewn and stapled gastrointestinal anastomoses. MAA relies on compression-mediated tissue fusion and delayed anastomotic maturation, whereas conventional techniques establish immediate luminal continuity through direct tissue approximation [7,11-13,18-20,22].

Feature	Magnet-assisted anastomosis	Hand-sewn anastomosis	Stapled anastomosis
Mechanism of anastomosis formation	Paired magnets generate controlled circumferential compression leading to localized ischemia, tissue necrosis, and gradual biologic fusion of bowel walls over time [7,13,20]	Sutures approximate tissue edges directly, allowing immediate tissue apposition and healing through standard wound repair mechanisms	Mechanical staples compress and approximate tissue edges immediately, producing instant luminal continuity
Time to functional luminal continuity	Delayed; anastomotic channel forms gradually over days to weeks during tissue remodeling and magnet compression	Immediate restoration of luminal continuity following suturing	Immediate restoration of luminal continuity following stapling
Foreign material at the anastomotic site	Temporary; magnets are expelled through the GI tract after anastomotic maturation, leaving no permanent implant	Permanent non-absorbable or slowly absorbable sutures remain in situ	Permanent metallic staples remain in situ
Technical requirements	Requires precise magnet positioning, adequate tissue alignment, and stable compression; may require endoscopic or fluoroscopic guidance during deployment	Requires advanced surgical suturing skill and careful tissue handling; technically demanding in minimally invasive settings	Mechanically facilitated; generally faster and more standardized once the staple device is positioned correctly
Dependence on tissue alignment and mechanical tension	Highly dependent on symmetric compression, luminal geometry, and tissue alignment; malposition may result in incomplete fusion or stricture formation [19,20,22]	Moderate dependence on tissue tension and perfusion; experienced surgeons can adjust technique intraoperatively	Moderate dependence on tissue thickness and staple height selection; staple line integrity is critical
Perioperative management	Delayed enteral feeding may be required until anastomosis forms; postoperative surveillance for magnet position and device expulsion is recommended	Enteral feeding is often resumed early, depending on the operative context and tissue integrity	Early enteral feeding is commonly feasible; staple line reinforcement is sometimes used in high-risk tissues
Early clinical safety observations	Early feasibility studies report successful magnet deployment and anastomosis formation with few device-related adverse events in small cohorts [12,13]	Established safety profile with known risks, including anastomotic leak, bleeding, and infection	Established safety profile with risks, including leak, bleeding, and staple misfire
Anastomotic leak risk	Limited human data; theoretical reduction due to circumferential compression and biologic fusion, but not yet demonstrated in comparative trials	Well-characterized risk depending on tissue perfusion, tension, and surgical technique	Well-characterized risk; staple line failure or ischemia may contribute to leaks
Long-term durability	Currently uncertain; limited longitudinal clinical data; outcomes likely influenced by alignment, compression force, and tissue remodeling	Long-term durability is well established across a wide range of GI procedures	Long-term durability is well established with predictable performance in most surgical contexts
Current evidence base	Small feasibility cohorts, early clinical studies, and limited preclinical investigations; comparative trials lacking	Extensive clinical experience and a large evidence base across surgical disciplines	Extensive clinical experience with substantial comparative data versus hand-sewn techniques
Clinical adoption	Investigational; primarily evaluated in bariatric and select pediatric or hepatobiliary contexts	Standard surgical technique used globally	Widely adopted standard technique in open and minimally invasive surgery
Potential advantages	Avoids permanent implants; may simplify reconstruction in confined minimally invasive spaces; biologic fusion may reduce foreign-body reaction	Flexible technique adaptable to complex anatomy	Rapid, reproducible, and widely standardized with specialized stapling devices

Current limitations and knowledge gaps

Despite ongoing technical exploration of MAA, its translation into routine GI surgical practice remains limited. The existing literature is dominated by preclinical investigations, case reports, and small early-phase clinical series, with a notable absence of adequately powered comparative trials evaluating MAA in common GI procedures such as colorectal resection or gastrojejunostomy using standardized definitions of anastomotic complications [7,12,13]. Consequently, meaningful conclusions regarding comparative safety, anastomotic leak rates, stricture formation, and long-term durability relative to conventional techniques cannot currently be established.

A fundamental limitation of MAA relates to its reliance on delayed anastomotic formation. In contrast to conventional techniques that establish immediate luminal continuity, magnetic compression induces progressive ischemia, tissue necrosis, and subsequent biologic fusion over a period of several days to weeks [19,20]. This delayed-healing paradigm introduces uncertainty regarding optimal perioperative management, particularly with respect to nutritional strategies, the potential need for proximal diversion, and the timing of radiographic or endoscopic confirmation of anastomotic patency. These parameters remain inconsistently described and poorly standardized across existing reports [7,13]. Additionally, the interval between tissue necrosis and complete fusion raises theoretical concerns regarding occult microleakage or bacterial translocation during the early postoperative period. To date, however, these risks have not been systematically evaluated in available clinical series.

Technical variability further complicates the interpretation of the existing literature. Successful MAA formation depends on accurate magnet alignment, appropriate compressive force, and uniform circumferential tissue apposition. However, currently reported studies employ heterogeneous magnet designs, sizes, and delivery platforms, and no consensus has been established regarding optimal compression parameters or standardized deployment techniques [19,20]. Excessive compressive force may cause unintended tissue injury beyond the anastomotic interface, whereas insufficient compression may lead to incomplete necrosis and anastomotic failure. Moreover, most currently available systems lack real-time feedback mechanisms capable of assessing tissue compression or perfusion during deployment, limiting the surgeon's ability to optimize anastomotic conditions intraoperatively and monitor compression dynamics postoperatively [19,20]. The learning curve associated with endoscopic, laparoscopic, or hybrid magnet placement has also not been systematically evaluated, raising additional questions regarding reproducibility outside specialized centers.

Long-term outcomes remain incompletely characterized. Most available reports describe short- to mid-term follow-up, with limited data addressing late complications such as anastomotic strictures, altered motility, changes in tissue compliance, or long-term functional integrity of the reconstructed segment [12,13]. Given the distinct healing mechanism of compression-induced necrosis followed by tissue remodeling, it remains uncertain whether long-term durability is equivalent to that achieved with conventional anastomotic techniques. In addition, patient-reported outcomes and quality-of-life measures remain largely absent from the existing literature.

Taken together, these limitations underscore that MAA remains an exploratory reconstructive strategy rather than an established surgical platform. Although the concept has generated growing technical investigation across multiple experimental and clinical contexts, the field remains fragmented, with limited methodological standardization and sparse comparative evidence. For this reason, the present review aims not to advocate for clinical adoption but rather to critically synthesize the current literature, clarify existing uncertainties, and identify the key scientific and clinical questions that must be addressed before the technology can be meaningfully evaluated in routine GI surgery.

Future directions 

Although experimental and early clinical investigations have explored the feasibility of MAA, the current evidence base remains limited, and substantial knowledge gaps must be addressed before its clinical role can be meaningfully evaluated. Foremost among these is the absence of high-quality comparative evidence. Well-designed multicenter randomized controlled trials comparing MAA with conventional sutured and stapled anastomoses across relevant GI procedures would be necessary to determine relative safety, efficacy, and durability. Such studies should incorporate standardized definitions of anastomotic complications, including leaks, strictures, bleeding, and reinterventions.

Long-term outcome data are also limited. Future investigations should assess not only early postoperative outcomes but also long-term anastomotic integrity, stricture formation, functional performance, and patient-reported quality-of-life measures. These outcomes are particularly important given the delayed mechanism of anastomotic formation associated with magnetic compression, which differs fundamentally from the immediate tissue approximation achieved with conventional techniques and may influence healing dynamics and late complications.

Patient selection criteria also remain poorly defined. Additional research is needed to identify anatomical, pathological, and physiological factors that may influence outcomes following MAA, including tissue perfusion, local inflammation, contamination, and nutritional status. Improved characterization of these variables may help clarify potential indications, contraindications, and risk stratification strategies should the technique undergo further clinical evaluation.

From a technical standpoint, ongoing device development may address several limitations identified in early studies. Potential areas of innovation include improved magnet delivery systems, refinements to compression profiles, and the development of devices that provide more consistent alignment and tissue apposition. Integration with advanced endoscopic or minimally invasive platforms may also facilitate procedural standardization if future studies demonstrate clinical utility.

Finally, broader health system considerations warrant evaluation. Formal cost analyses comparing MAA with established anastomotic methods should consider device costs, operative duration, complication rates, length of hospital stay, and downstream healthcare utilization. In addition, the implications for surgical training, procedural learning curves, and reproducibility across centers remain unclear and would require systematic investigation.

Taken together, these research priorities highlight the exploratory status of MAA within GI surgery. Whether magnetic compression techniques will ultimately identify a defined clinical role or remain a limited investigational concept will depend on the results of rigorous comparative studies and long-term outcome evaluations.

## Conclusions

MAA represents a mechanistically distinct approach to GI reconstruction that relies on controlled tissue compression, ischemia, and subsequent biologic fusion rather than immediate mechanical approximation with sutures or staples. Early clinical and experimental investigations demonstrate that magnetic compression can achieve technically successful anastomotic formation in carefully selected settings. However, the available evidence remains limited to small exploratory cohorts and heterogeneous procedural applications, with relatively short follow-up and minimal comparative evaluation against established reconstructive techniques. Importantly, the current literature highlights several recurring barriers that have historically limited the broader adoption of compression-mediated anastomosis. These include the delayed nature of anastomotic maturation, dependence on precise tissue alignment and compression parameters, variability in device design and deployment techniques, and the absence of standardized perioperative management strategies. Together, these factors complicate reproducibility and hinder meaningful comparison with conventional stapled or hand-sewn methods. The present review, therefore, contributes not by advocating clinical adoption but by critically synthesizing the fragmented body of existing evidence and identifying the key scientific and technical questions that must be addressed before the technology can be rigorously evaluated. In particular, the field requires standardized device platforms, consistent reporting of anastomotic outcomes, and adequately powered comparative studies assessing long-term durability, complication profiles, and functional performance. Until such evidence becomes available, MAA should be regarded as an investigational reconstructive strategy rather than a validated alternative to conventional GI anastomotic techniques. Whether magnetic compression ultimately identifies a defined role in GI surgery or remains a specialized experimental approach will depend on the outcomes of future systematic clinical evaluation.
